# Effect of Biofumigation on Population Densities of *Pratylenchus* spp. and *Verticillium* spp. and Potato Yield in Eastern Canada

**DOI:** 10.1007/s12230-022-09875-2

**Published:** 2022-04-13

**Authors:** Dahu Chen, Bernie J. Zebarth, Claudia Goyer, Louis-Pierre Comeau, Kamrun Nahar, Tom Dixon

**Affiliations:** 1 Fredericton Research and Development Centre, Agriculture and Agri-Food Canada, 95 Innovation Road, Fredericton, New Brunswick E3B 4Z7 Canada; 2 McCain Foods (Canada) Ltd., 8800 Main Street, Florenceville-Bristol, New Brunswick E7L 1B2 Canada

**Keywords:** Root lesion nematodes, Verticillium wilt, *Solanum tuberosum*, Crop rotation, Fumigation, Potato early dying

## Abstract

Biofumigation has been proposed as an alternative to soil fumigation to manage soil-borne diseases including potato early dying disease complex (PED). This study examined the potential of using brown mustard (*Mustard juncea*) biofumigation to manage PED under rain-fed potato production in New Brunswick, Canada in two trials between 2017 and 2020 in comparison with chloropicrin fumigation and a conventional barley rotation. Biofumigation increased yield in one trial, but not in a second trial where the potato crop experienced severe drought, whereas chloropicrin fumigation increased yield in both trials. Biofumigation was effective in suppressing root-lesion nematode (RLN, *Pratylenchus* spp.) counts in both trials, but was ineffective in suppressing *V. dahliae* population density. Chloropicrin fumigation was effective in suppressing RLN counts and *V. dahliae* population density only in the hill where injected, but the effect was short-lived as the population density of *V. dahliae* in the hill increased to the level of the control in one potato growing season. Biofumigation may be an alternative to chloropicrin fumigation in managing PED, particularly in fields with high RLN population but relatively low *Verticillium* population density. However, neither biofumigation nor fumigation used alone may be sustainable in the short-term potato rotations commonly used in New Brunswick, and additional beneficial practices are required to sustain productivity in the long-term.

## Resumen

La biofumigación se ha propuesto como una alternativa a la fumigación del suelo para manejar las enfermedades transmitidas por el suelo, incluido el complejo de enfermedades de muerte prematura de la papa (PED). Este estudio examinó el potencial del uso de la biofumigación de mostaza marrón (*Mustard juncea*) para manejar la PED bajo la producción de papa de secano en New Brunswick, Canadá, en dos ensayos entre 2017 y 2020 en comparación con la fumigación con cloropicrina y una rotación de cebada convencional. La biofumigación aumentó el rendimiento en un ensayo, pero no en un segundo ensayo en el que el cultivo de papa experimentó una sequía severa, mientras que la fumigación con cloropicrina aumentó el rendimiento en ambos ensayos. La biofumigación fue efectiva para suprimir los conteos del nematodo lesionador de la raíz (RLN, *Pratylenchus* spp.) en ambos ensayos, pero fue ineficaz para suprimir la densidad de población de *V. dahliae*. La fumigación con cloropicrina fue efectiva para suprimir los conteos de RLN y la densidad de población de *V. dahliae* solo en el lomo del surco donde se inyectó, pero el efecto fue de corta duración ya que la densidad de población de *V. dahliae* en el surco aumentó al nivel del testigo en un ciclo de cultivo de papa. La biofumigación puede ser una alternativa a la fumigación con cloropicrina en el manejo de la PED, particularmente en campos con alta población de RLN pero densidad de población de *Verticillium r*elativamente baja. Sin embargo, ni la biofumigación ni la fumigación utilizadas por sí solas pueden ser sustentables en las rotaciones de papa a corto plazo comúnmente utilizadas en New Brunswick, y se requieren prácticas benéficas adicionales para mantener la productividad a largo plazo.

## Introduction

Potato (*S. tuberosum* L.) is the most important vegetable crop grown in Canada, and Eastern Canada accounted for 51% of national potato production in 2017 (Agriculture and Agri-Food Canada, 2019). Despite technological advances, only limited improvements in potato yield have been observed in Eastern Canada in recent decades (Wilson et al., 2018; Zebarth et al., 2021). This lack of yield improvement has been attributed to declining soil organic matter, increasing soil-borne disease pressure and climate change in the rain-fed potato production systems of this region (Dahal et al., 2019; Nyiraneza et al., 2021; Wilson et al., 2018). In particular, potato early dying (PED) disease complex, which can cause 30 to 50% potato yield reduction in severely affected fields of North America (Davis et al., 2001; Powelson & Rowe, 1993), is believed to be an important yield limiting factor.

The primary pathogen of PED is *Verticillium* spp., and the presence of root-lesion nematodes (RLN, *Pratylenchus* spp.) exacerbates the disease severity and yield loss (Kimpinski & Thompson, 1990; Powelson & Rowe, 1993). *V. albo-atrum* was previously reported to be the main species in Eastern Canada causing potato wilt (Easton et al., 1972; Kimpinski et al., 1998; Robinson, 1961), however currently, *V. dahliae* has become prevalent (Borza et al., 2018). Three RLN species were identified in Eastern Canada (Yu, 2008), and two of them (*Pratylenchus penetrans* and *P. crenatus*) were widespread (Kimpinski et al., 1998; Kimpinski & Thompson, 1990). *P. penetrans* often interacts with *V*. *dahliae*, causing severe PED, while *P. crenatus* does not (Bowers et al., 1996; Orlando et al., 2020). Recent surveys of potato fields in New Brunswick (NB) and Prince Edward Island (PEI) found high population densities of *V. dahliae* and *Pratylenchus* spp., and severe PED disease symptoms, in a majority of potato fields (unpublished data).

Control of PED is extremely difficult due to the characteristics of the pathogens for their broad host ranges and long soil survival ability (Bélair et al., 2007; Kimpinski & Dunn, 1985; Townshend & Davidson, 1960; Woolliams, 1966), as well as lack of high PED resistant potato cultivars. The most effective approach to control soil-borne diseases including PED is to use soil fumigation (e.g., chloropicrin or metam sodium) either alone or in combination with other strategies (Ben-Yephet et al., 1983; Davis et al., 1986; Powelson & Rowe, 1993; Taylor et al., 2005; Tsror et al., 2005). In recent years, there has been more interest in soil fumigation by some potato growers in Canada, and field trials were conducted to examine the potential to reduce PED and other soil-borne diseases and to improve potato yield (Al-Mughrabi et al., 2016; Halsall, 2016; Molina et al., 2014). However, concerns over potential negative effects of chemical fumigants on the environment, human health, and soil health (Bünemann et al., 2006; Sande et al., 2011), as well as the high cost of applying fumigation, has increased interest in identifying alternative strategies to chemical fumigation for PED management.

One promising alternative to chemical fumigation is biofumigation. Biofumigation uses glucosinolate-containing cruciferous crop residues to control soil-borne diseases and pests by volatile compounds toxic to microorganisms (Kirkegaard et al., 1993; Larkin et al., 2011; Larkin & Griffin, 2007; McGuire, 2003). Success of biofumigation is affected by many factors, including the glucosinolate content in plant tissues, the amount of plant biomass incorporated, the speed and effectiveness of biomass incorporation, and the environmental conditions (Mattner et al., 2008; Kruger et al., 2013). Mustard (*Brassica* spp.) as a rotation crop, or applied as seed-meal, has been demonstrated to reduce PED and other soil-borne diseases of potato (Larkin et al., 2011; Larkin & Griffin, 2007; Molina et al., 2014; Ngala et al., 2015). The Caliente mustard (e.g., Caliente 119, Caliente 199) (*B. juncea* and *Sinapis alba* blend) were bred specifically for biofumigation and green manuring, and were demonstrated to reduce Verticillium wilt and other soil-borne diseases (Larkin et al., 2011; Larkin & Halloran, 2014; Wang et al., 2009), but their seeds are more costly than conventional mustard varieties. The ‘Centennial Brown’ brown mustard (*B. juncea* (L.) Czern.) has a greater glucosinolate content than the common brown mustard (Rakow et al., 2009) and the seed is more affordable than for the Caliente mustard. Limited information is available on the potential of using mustard as a biofumigant crop for control of PED under rain-fed potato production systems in Eastern Canada.

The objective of this study was to evaluate the effect of biofumigation on the population density of *Pratylenchus* spp. and *Verticillium* spp., PED severity and potato tuber yield under rain-fed potato production in Eastern Canada. New Brunswick was selected as an experimental site to conduct the experiment for its geographic representation, high economic importance of the potato industry, and regulatory permission to conduct soil fumigation with chloropicrin. The potential of using ‘Centennial Brown’ brown mustard as a biofumigant crop to control PED was examined in two trials in commercial fields in New Brunswick, where each trial was conducted over a two-year rotation (2017–2018 and 2019–2020). Biofumigation was compared with spring barley plus fall chloropicrin fumigation as a positive control and a conventional spring barley as a negative control.

## Materials and Methods

### Field Sites and Experimental Treatments

Two field trials were conducted in commercial fields in NB with a known history of PED. Both fields were owned and operated by the same potato grower and had the same standard farm practices. Trial A (2017–2018) was located in Jacksonville, NB and Trial B (2019–2020) in Wilmot, NB, located approximately 10 and 20 km from Woodstock, NB, respectively. Trial A had never been fumigated prior to this experiment whereas Trial B had been fumigated with chloropicrin in 2017 for control of PED. Soils at the experimental sites had a loam soil texture. Soil pH (1:1 water) averaged 5.9 and 6.0 and soil total carbon (dry combustion) averaged 18.5 and 21.8 g kg^−1^ for Trials A and B, respectively, for 0–25 cm soil depth. Climate data was obtained from the Environment and Climate Change Canada weather station at Woodstock, NB (https://climate.weather.gc.ca/historical_data/search_historic_data_e.html).

**Fig. 1:**
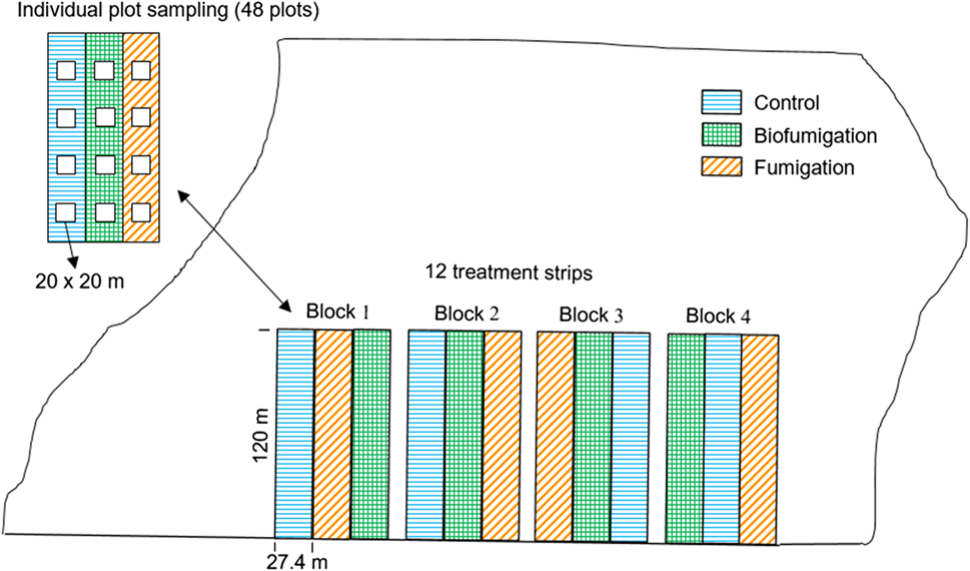
Schematic diagram of the field plot layout in trial A showing the three treatments (control, biofumigation and fumigation) in four blocks and 48 individual plots for sampling. A similar design was used for trial B. Dimensions are not to scale

The experiment used a randomized complete block design with three treatments and four blocks (Fig. 1). The experimental unit was a treatment strip (27.4 m wide by approximately 120 m long), and each treatment strip had four sampling plots (20 × 20 m in size) to capture the spatial variability within the field. Each trial had a two-year crop sequence with experimental treatments established in the rotation crop phase (2017 and 2019) and potatoes grown in the subsequent year (2018 and 2020). Treatments included: 1) control (i.e.*,* no fumigation or biofumigation), seeded to spring barley cultivar ‘Leader’ (*Hordeum vulgare* L.); 2) fumigation, seeded to spring barley cultivar ‘Leader’ followed by a fall chloropicrin application; and 3) biofumigation, seeded to two consecutive crops during the growing season of a high glucosinolate brown mustard cultivar ‘Centennial Brown’ (Speare Seeds Limited, Ontario).

For the control and fumigation treatments, spring barley was seeded on 19 May 2017 and 2 June 2019 for trials A and B, respectively, at a seeding rate of 140 kg ha^−1^ and harvested in late August. For the biofumigation treatment, the first crop of mustard was seeded on 16 June 2017 and 24 June 2019 at a seeding rate of 11.2 kg ha^−1^ according to seed company recommendation. The mustard plants were mowed and then immediately incorporated into the soil with disking on 20 July 2017 and 31 July 2019 at full flowering (i.e., approximately 50% flowers in main raceme open, older petals falling) for greatest biofumigation potential (Doheny-Adams et al., 2018; Saskatchewan mustard development commission., 2021). The second crop of mustard was seeded on 3 August 2017 and 6 August 2019 and mowed and immediately incorporated into the soil on 20 September 2017 and 14 September 2019 for additional biomass and biofumigation enhancement. The dry biomass of the rotation crops in 2017 was estimated by hand sampling all above-ground plant tissue from a 1 m^2^ quadrat in each plot on 19 July and 20 September, 2017 for mustard before incorporation and on 31 July 2017 for barley before harvest. Mustard produced 3.8 and 2.4 Mg ha^−1^ of dry biomass for the first crop and the second crop, respectively. Barley produced 9.6 Mg ha^−1^ of dry biomass in the control and fumigation treatments, respectively, however, only the straw was incorporated into the soil. The entire experimental site was fall plowed and hills (i.e., ridges to be used for planting potatoes in the following growing season) were formed in late September. Chloropicrin (TriEst Ag Group, Inc.) was injected into the hills at 129 kg ha^−1^ according to standard practice. While hills would not normally be formed at this time for the control and biofumigation treatments, hills were formed in this experiment to allow for direct comparison with the fumigation treatment. Potato variety ‘Russet Burbank’ was planted on 14 May 2018 and 18 May 2020 following standard grower practices. Potato tuber yield was determined in late September in both years by manually harvesting two 3-m rows in the middle of each sampling plot. Total yield, marketable yield and tuber specific gravity were assessed by McCain Foods (Canada) Ltd. according to the processing contract specifications.

### Potato Early Dying Severity Assessment

Disease severity of PED was assessed in Trial A in 2018. The percentage of foliar wilt (chlorosis and necrosis) was rated from 15 plants in each of two rows in each sampling plot four times between 3 August and 19 September (MacGuidwin & Rouse, 1990). The area under disease progress curve (AUDPC) was calculated to determine the disease severity (Shaner & Finney, 1977). For Trial B in 2020, due to the COVID-19 travel restrictions, only one disease rating was made on 3 September 2020.

### Soil Sampling

One composite soil sample was collected from 0 to 25 cm depth in each sampling plot in spring before planting rotation crops (i.e., 2017 and 2019) using a Dutch augur where each composite soil sample consisted of five cores obtained using a random sampling pattern. The soil was mixed thoroughly and a total of approximately 2 kg soil was retained for each composite soil sample. In fall of the rotation crop phase, one composite sample was collected separately in the hill and in the furrow (i.e., space between the hills) from each sampling plot in October, approximately two weeks after soil fumigation, as described above. In the potato phase (i.e.*,* 2018 and 2020), one composite soil sample was collected separately in the hill and in the furrow in each sampling plot in spring after planting, and in fall at harvest in 2018, whereas one composite soil sample was collected in the hill only in each sampling plot at harvest in 2020 due to COVID-19 travel restriction and labor shortage.

The soil was kept cool during sampling and transportation before being stored at 4 °C. Approximately 500 g each soil sample was sent to a commercial laboratory for RLN counts within one week after sampling. The remainder of the soil was air-dried and sent to a commercial laboratory for *Verticillium* DNA quantification.

### Root-Lesion Nematodes and *Verticillium dahliae* Quantification

Root-lesion nematodes (*Pratylenchus* spp.) in all composite soil samples were quantified at Agriculture & Food Laboratory Services in University of Guelph based on the Baermann pan method for extraction of nematodes from soil (Townshend, 1963), except for the samples from the furrow in fall 2018 which were stored too long before a reliable quantification could be obtained.

*V. dahliae* population densities were determined in all samples in Trial A and Trial B, while *V. albo-atrum* population densities were determined in all 2018 samples in Trial A and all samples in Trial B. *V. tricorpus* population densities were determined in all samples in Trial B.

Soil (300 g) was well mixed, air-dried and passed through a 2-mm sieve. A subsample (50 g) of soil was ground to a fine powder using a grinding mill. Three 0.25 g samples of soil were extracted separately from each composite sample using EZNA® Soil DNA Kit (Omega Bio-tek; cat#D5625) according to the manufacturer protocol. Extraction success and cross-contamination control were assessed by extraction of known clean, sterile soil with (positive control) and without (negative control) the addition of a few grains (~0.1 mg) of dried, powdered fungal material scraped from pure culture plates of *V. dahliae and V. albo-atrum*. Total soil DNA extracted was measured by a spectrophotometer (Eppendorf BioPhotometer, Germany).

Concentrations of *V. dahliae*, *V. albo-atrum* and *V. tricorpus* DNA in the samples were quantified by probe-based quantitative real-time PCR (qPCR) using primers and probes targeting the *ef1α* gene of each species by Agricultural Certification Services Inc. (Fredericton, NB, Canada) as detailed in Nyiraneza et al. (2021).

### Data Treatment and Statistic Analysis

Population densities of the RLN and *V. dahliae*, disease severity and potato yield and tuber specific gravity were subjected to two-way ANOVA analysis. Post-hoc Tukey’s honest significant difference test was used to compare among treatment means. Log(100 + x) was used to transform the RLN counts and *Verticillium* DNA to meet the requirements of the ANOVA (Vrain et al., 1996). Linear regression analysis was performed to examine the relationship of pathogen densities and PED severity with tuber yield. All statistical analyses were performed using SigmaPlot 14.5 (Systat Software, Inc).

## Results

### Climatic Conditions

Average growing season (May to September) air temperature was 103, 104, 95 and 99% of the long-term (1981–2010) average of 15.6 °C for 2017 to 2020, respectively, and growing season total precipitation was 70, 94, 81 and 36% of the long-term average of 482 mm in 2017 to 2020, respectively (Table 1). As a result, relatively dry soil conditions were present in July and September of 2017 and in July of 2019 when mustard crops were incorporated. The exceptionally dry conditions in 2020 resulted in poor potato crop growth.

**Table 1:** Average daily mean air temperature and total precipitation during the May to September growing season in Woodstock, New Brunswick between 2017 and 2020 in comparison with the long-term (1981–2010) average. Climate data was obtained from the Environment and Climate Change Canada weather station at Woodstock, NB (https://climate.weather.gc.ca/historical_data/search_historic_data_e.html)

	Average air temperature (°C)					Total precipitation (mm)				
Month	2017	2018	2019	2020	1981–2010	2017	2018	2019	2020	1981–2010
May	10.9	11.9	8.9	8.3	10.9	133.4	80.0	58.2	61.8	94.2
June	16.3	14.3	15.2	16.7	16.3	68.8	107.4	111.6	28.6	91.0
July	18.7	21.1	19.9	20.3	19.0	37.8	97.2	37.2	27.1	100.2
Aug	17.8	19.8	17.7	18.6	18.4	38.0	109.6	85.6	36.0	100.6
Sept	16.4	14.0	12.3	13.5	13.2	57.2	57.6	97.0	18.9	95.7
Average or total	16.0	16.2	14.8	15.5	15.6	335.2	451.8	389.6	172.4	481.7

### Root-Lesion Nematode Population Density

Root-lesion nematodes were detected in all samples. As would be expected, no significant differences in the RLN density were detected among the treatments at the initiation of rotation crops in spring 2017 for Trial A (Table 2) and in spring 2019 for Trial B (Table 3).

**Table 2:** Population density (kg^−1^of dry soil) of root-lesion nematodes (*Pratylenchus* spp.) in Trial A measured on four dates: spring prior to planting rotation crops; in fall after treatments in 2017; right after planting potato; and at potato harvest in fall of 2018

	2017 spring	2017 fall			2018 spring			2018 fall	
Treatment		Hill	Furrow	Average	Hill	Furrow	Average	Hill	Furrow
Control	1254 a	1146 a	1519 a	1333 a	1486 a	1430 a	1458 a	4113 a	ND
Biofumigation	1385 a	1043 a	953 b	998 b	678 b	849 b	763 b	2075 b	ND
Fumigation	1076 a	321 b	1344 ab	833 b	379 c	1203 ab	791 b	1426 b	ND
Standard error of the mean	131	100	148	96	112	160	104	343	ND
Significance of ANOVA	NS	***	*	*	***	*	***	***	ND

Values followed by the same letter within a column are not significant different. NS, not significant at 5% probability level; *, *** significant at 0.05 and 0.001 probability levels, respectively. ND: not determined

**Table 3:** Population density (kg^−1^ of dry soil) of root-lesion nematodes (*Pratylenchus* spp.) in Trial B measured on four dates: spring prior to planting rotation crops; in fall after treatments in 2019; right after planting potato; and at potato harvest in fall of 2020

	2019 spring	2019 fall			2020 spring			2020 fall	
Treatment		Hill	Furrow	Average	Hill	Furrow	Average	Hill	Furrow
Control	685 a	870 a	1573 ab	1221 a	128 a	396 b	262 ab	619 a	ND
Biofumigation	491 a	509 b	1171 b	840 a	57 b	202 b	129 b	152 b	ND
Fumigation	563 a	76 c	2100 a	1088 a	29 b	708 a	368 a	143 b	ND
Standard error of the mean	86	87	215	120	18	85	45	72	ND
Significance of ANOVA	NS	***	*	NS	**	**	**	***	ND

Values followed by the same letter within a column are not significant different. NS, not significant at 5% probability level; *, **, *** significant at 0.05, 0.01 and 0.001 probability levels, respectively. ND: not determined

The RLN counts in the control treatment were generally stable over the growing season during the rotation phase in Trial A, but increased in Trial B (Tables 2 and 3). Compared with the control, RLN counts in the biofumigation treatment were significantly (37%) lower in the furrow but not the hill in Trial A, and significantly (42%) lower in the hill but not the furrow in Trial B, at the end of the growing season in the rotation phase. In contrast, fumigation resulted in a substantial decrease in RLN counts in the hill compared with the control (72 and 91% in Trials A and B, respectively), but did not significantly reduce RLN counts in the furrow, at the end of the growing season. The RLN averaged across the hill and furrow were significantly reduced by 25% and 38% in the biofumigation and fumigation treatments, respectively, compared to the control in fall 2017, but did not differ from the control in fall 2019.

In the potato phase, RLN counts in the control treatment prior to planting were generally similar to RLN counts the previous fall in Trial A, but lower than in the previous fall for Trial B (Tables 2 and 3). Compared with the control, RLN prior to potato planting in the biofumigation treatment were significantly lower in both the hill and furrow (55 and 41%, respectively) in Trial A, and significantly (56%) lower in the hill but not the furrow in Trial B. For the fumigation treatment at this time, RLN counts prior to potato planting were significantly (74%) lower compared with the control in the hill, but not the furrow in Trial A, whereas in Trial B, RLN counts for the fumigation treatment were significantly (77%) lower in the hill, but significantly (79%) greater in the furrow. The RLN averaged across the hill and furrow were significantly reduced by 48% and 46% in the biofumigation and fumigation treatments in spring 2018, respectively, compared to the control, but did not differ from the control in spring 2020.

The RLN counts increased over the potato growing season, particularly in Trial A (Tables 2 and 3). At potato harvest, RLN counts in the hill in the biofumigation treatment were significantly lower in the hill compared with the control in both trials (50 and 75% in Trials A and B, respectively). Similarly, RLN counts in the hill at harvest for the fumigation treatment were lower in both trials (65 and 77% in Trials A and B, respectively) compared with the control, and were not significantly different than RLN counts for the biofumigation treatment.

Overall, the RLN population densities were numerically 1.8 to 2.8 times greater in the spring of the rotation phase in Trial A compared with Trial B, and were 6.6 to 13.7 times greater in Trial A compared to Trial B at potato harvest.

#### *Verticillium* spp. Population Density

In the rotation phase, *V. dahliae* was detected in 90 and 92% of the spring and fall samples, respectively, in 2017 in Trial A, and in 100% of the spring and fall samples in 2019 in Trial B. *V. albo-atrum* was not determined in Trial A samples, but it was detected in 44 and 29% of the spring and fall samples, respectively, in Trial B in 2019. *V. tricorpus* was not determined in Trial A samples, but it was not detectable in samples of Trial B in 2019.

In the potato phase, *V. dahliae* was detected in 88 and 100% of the spring and fall samples, respectively, in 2018 in Trial A and in 95 and 100% of the spring and fall samples, respectively, in 2020 in Trial B. *V. albo-atrum* was detected in 7 and 5% of the spring and fall samples, respectively, in 2018, and in 15 and 48% of the spring and fall samples, respectively, in 2020. The detection of *V. albo-atrum* was sporadic since the quantity was near zero with only 1 of 3 (in 2018) or 1 of 6 (in 2019 and 2020) qPCR reactions showing positive reaction near the detection limit. No further analyses were done for *V. albo-atrum.* The *V. tricorpus* was not detectable in any samples in 2020.

Initial population densities of *V. dahliae* did not vary significantly among treatments in the spring prior to planting the rotation crops in 2017 (Table 4) and in 2019 (Table 5). In the fall of the rotation phase at two weeks after the time of fumigation, no significant differences in *V. dahliae* population density were detected among the treatments in either the hill or furrow, or average across the hill and furrow, in 2017 in Trial A (Table 4) or in the furrow in 2019 in Trial B (Table 5). In contrast, *V. dahliae* population density in the fall of 2019 in Trial B varied significantly among treatments, and followed the pattern biofumigation > control > fumigation (Table 5). When averaged across the hill and furrow, no difference was detected between the biofumigation and the control, while fumigation significantly reduced 31% *V. dahliae* population density compared to the control, in fall 2019.

**Table 4:** Population density (pg DNA g^−1^ of dry soil) of *Verticillium dahliae* in Trial A measured on four dates: spring prior to planting rotation crops and in fall after treatments in 2017; right after planting potato and at potato harvest in fall of 2018

	2017 spring	2017 fall			2018 spring			2018 fall		
Treatment		Hill	Furrow	Average	Hill	Furrow	Average	Hill	Furrow	Average
Control	25 a	31 a	36 a	33 a	150 a	136 a	143 a	547 a	300 a	423 a
Biofumigation	22 a	29 a	42 a	35 a	95 a	137 a	116 a	321 a	241 a	281 a
Fumigation	20 a	43 a	30 a	36 a	18 b	115 a	66 b	410 a	335 a	373 a
Standard error of the mean	4	6	8	6	15	17	12	78	35	44
Significance of ANOVA	NS	NS	NS	NS	***	NS	***	NS	NS	NS

Values followed by the same letter within a column are not significant different. NS, not significant at 5% probability level; *** significant at 0.001 probability level

**Table 5:** Population density (pg DNA g^−1^ of dry soil) of *Verticillium dahliae* in Trial B measured on four dates: spring prior to planting rotation crops and in fall after treatments in 2019; right after planting potato and at potato harvest in fall of 2020

	2019 spring	2019 fall			2020 spring			2020 fall	
Treatment		Hill	Furrow	Average	Hill	Furrow	Average	Hill	Furrow
Control	47 a	87 b	54 a	71 a	86 a	35 a	60 a	428 ab	ND
Biofumigation	52 a	116 a	36 a	76 a	99 a	24 a	62 a	475 a	ND
Fumigation	63 a	52 c	45 a	49 b	8 b	29 a	18 b	319 b	ND
Standard error of the mean	5	8	6	5	8	5	5	41	ND
Significance of ANOVA	NS	***	NS	***	***	NS	***	*	ND

Values followed by the same letter within a column are not significant different. NS, not significant at 5% probability level; *, *** significant at 0.05 and 0.001 probability levels, respectively. ND: not determined

In the spring in the potato phase, the fumigation treatment significantly reduced the *V. dahliae* population density compared with the control in the hill in both 2018 and 2020, but not in the furrow (Tables 4 and 5). In comparison, the *V. dahliae* population density for the biofumigation treatment did not differ significantly from the control in either the hill or furrow in the spring of either year. In fall at potato harvest, no significant differences in *V. dahliae* population density were detected among the treatments in either the hill or furrow or average over the hill and furrow in 2018 (Table 4), whereas *V. dahliae* population density was greater for biofumigation than for fumigation in the hill or average over the hill and furrow in 2020 (Table 5).

The increase in *V. dahliae* population density over the growing season in the rotation phase was generally small, ranging from 0.8- to 2.2-fold among the treatments in 2017 and 2019, respectively, however, the increase in population density was greater in the potato phase, ranging from 3- to 5-fold for the control and biofumigation and 23- to 40-fold for the fumigation treatment in 2018 and 2020, respectively. The initial population density of *V. dahliae* in Trial B in 2019 was approximately 2- to 3-fold greater than in Trial A in 2017 in the spring of the rotation phase. However at potato harvest, the *V. dahliae* population density in Trial B was 0.8- to 1.5-fold greater than for Trial A.

### Potato Early Dying Severity

**Fig. 2:**
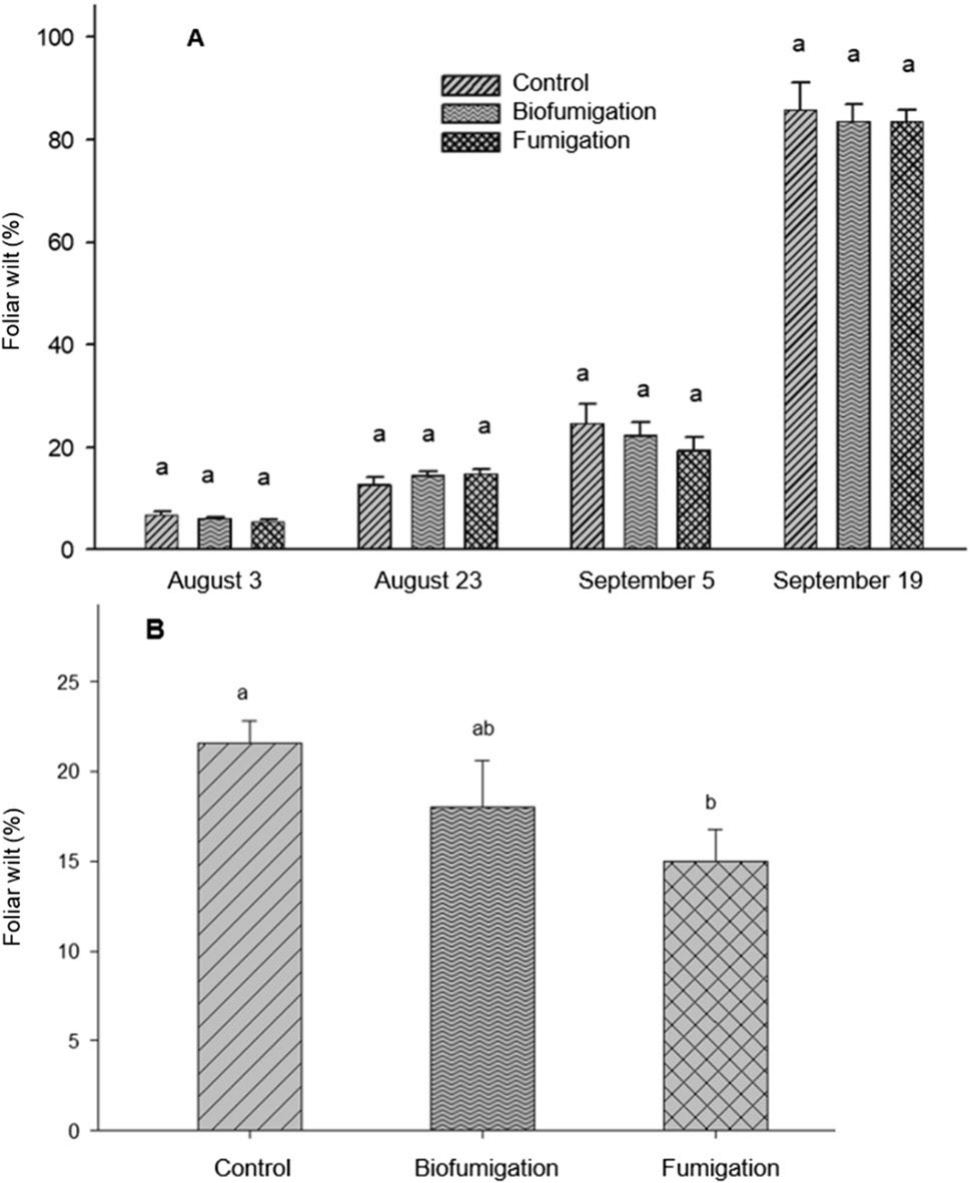
The percent of foliar wilt of potato early dying disease complex on potato variety ‘Russet Burbank’ rated on three dates between 3 August and 19 September 2018 **A** and on 3 September 2020 **B**. Bars with the same letter are not significant different at p ≤ 0.05

Visual symptoms of PED appeared in mid-July in both trials. The PED severity did not differ significantly among treatments over the monitoring period between early August and mid-September of 2018 in Trial A (Fig. 2A). The disease development based on the area under the disease progress curve (AUDPC) was similar among the treatments with the AUDPC being 1162, 1134, and 1092 for the control, biofumigation, and fumigation, respectively. The rating for foliar wilt in Trial B done on 3 September 2020 was significantly different among the treatments, ranging from 15% for the fumigation treatment to 22% for the control treatment (Fig. 2B).

### Potato Yield

Average total and marketable yield in 2018 (51.6 and 41.8 Mg ha^−1^, respectively) were much greater than in 2020 (30.7 and 27.5 Mg ha^−1^, respectively) (Table 6). In Trial A, the fumigation and biofumigation treatments significantly increased total and marketable yield by an average of 11 and 12%, respectively, compared to the control. In Trial B, the fumigation treatment increased total and marketable yield by 14 and 19%, respectively, compared with the control, whereas the biofumigation treatment did not significantly increase yield compared with the control. There was no significant effect of treatment on tuber specific gravity in Trial A, while in Trial B, the fumigation treatment significantly increased specific gravity compared with the control.

**Table 6:** Effect of the treatments on potato total and marketable yields and tuber specific gravity in Trials A and B

	Trial A (2018)			Trial B (2020)		
	Total yield	Marketable yield	Specific gravity	Total yield	Marketable yield	Specific gravity
Treatment	Mg ha^−1^			Mg ha^−1^		
Control	48.2 b	37.7 b	1.089 a	29.0 b	25.7 b	1.091 b
Biofumigation	52.2 a	41.2 a	1.090 a	30.0 b	26.3 b	1.093 ab
Fumigation	54.5 a	43.4 a	1.090 a	33.1 a	30.6 a	1.095 a
Standard error of the mean	1.0	1.0	0.001	0.6	1.0	0.001
Significance of ANOVA	***	**	NS	***	***	**

Values followed by the same letter within a column are not significant different. NS, not significant at 5% probability level; ** and *** significant at 0.01 and 0.001 probability levels, respectively

### Relationship between Pathogen Density, PED Severity and Potato Tuber Yield

**Fig. 3:**
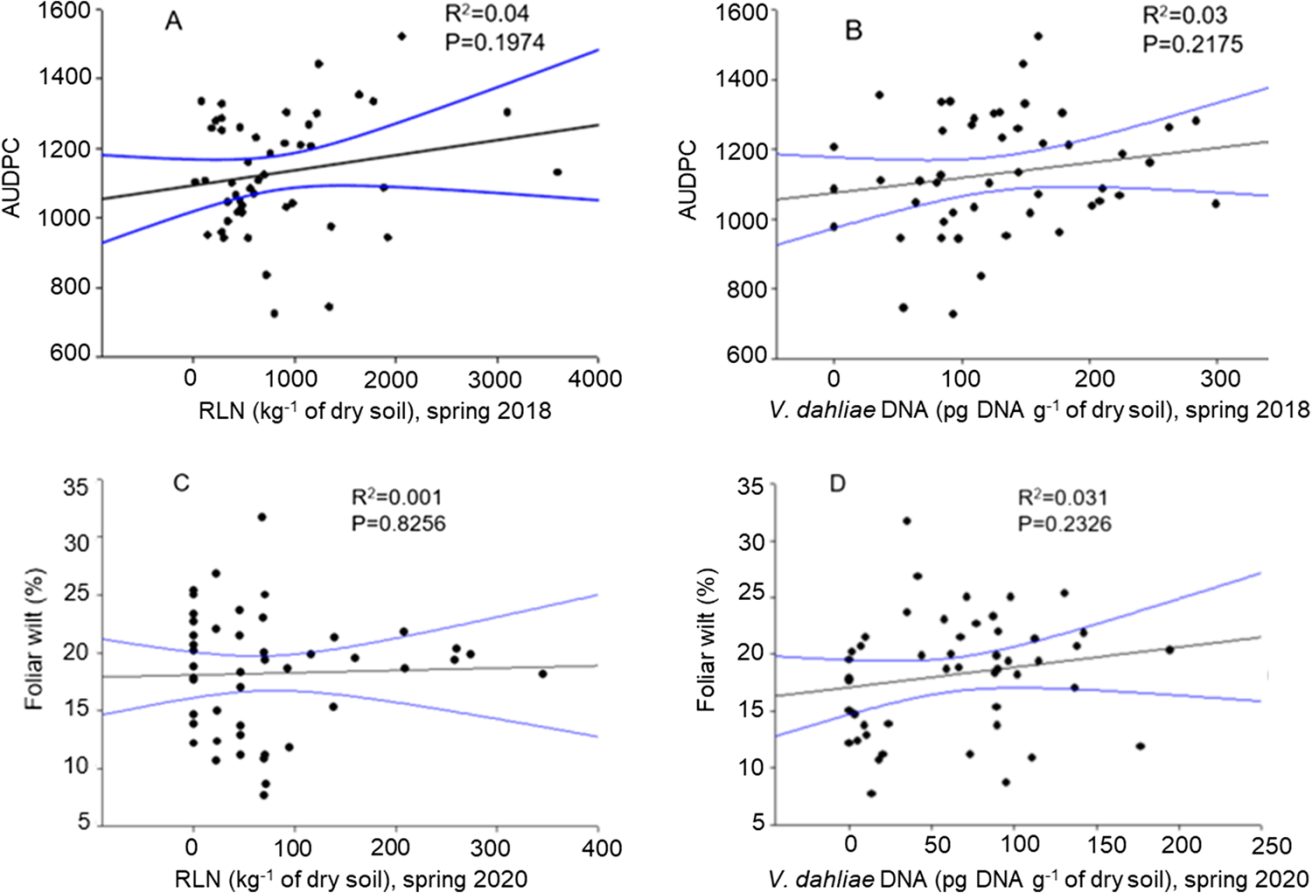
Relationship between the root-lesion nematode (RLN) density or the *Verticillium dahliae* density from samples taken from potato hill soon after planting in spring and the PED severity (AUDPC) **A, B** in 2018, or percent of foliar wilt **C, D** in 2020  95% confidence interval

The RLN and *V. dahliae* were present in all plots but varied in their densities in both trials. The incidence of the PED was 100% for both trials, and severity ranged from 59 to 99% foliar wilt of the plants at the last rating on 19 September 2018, and 7 to 32% on 3 September 2020. Both RLN and *V. dahliae* population densities were not significantly correlated with the PED severity as expressed in AUDPC in 2018 or in percent foliar wilt in 2020 (Fig. 3).

**Fig. 4:**
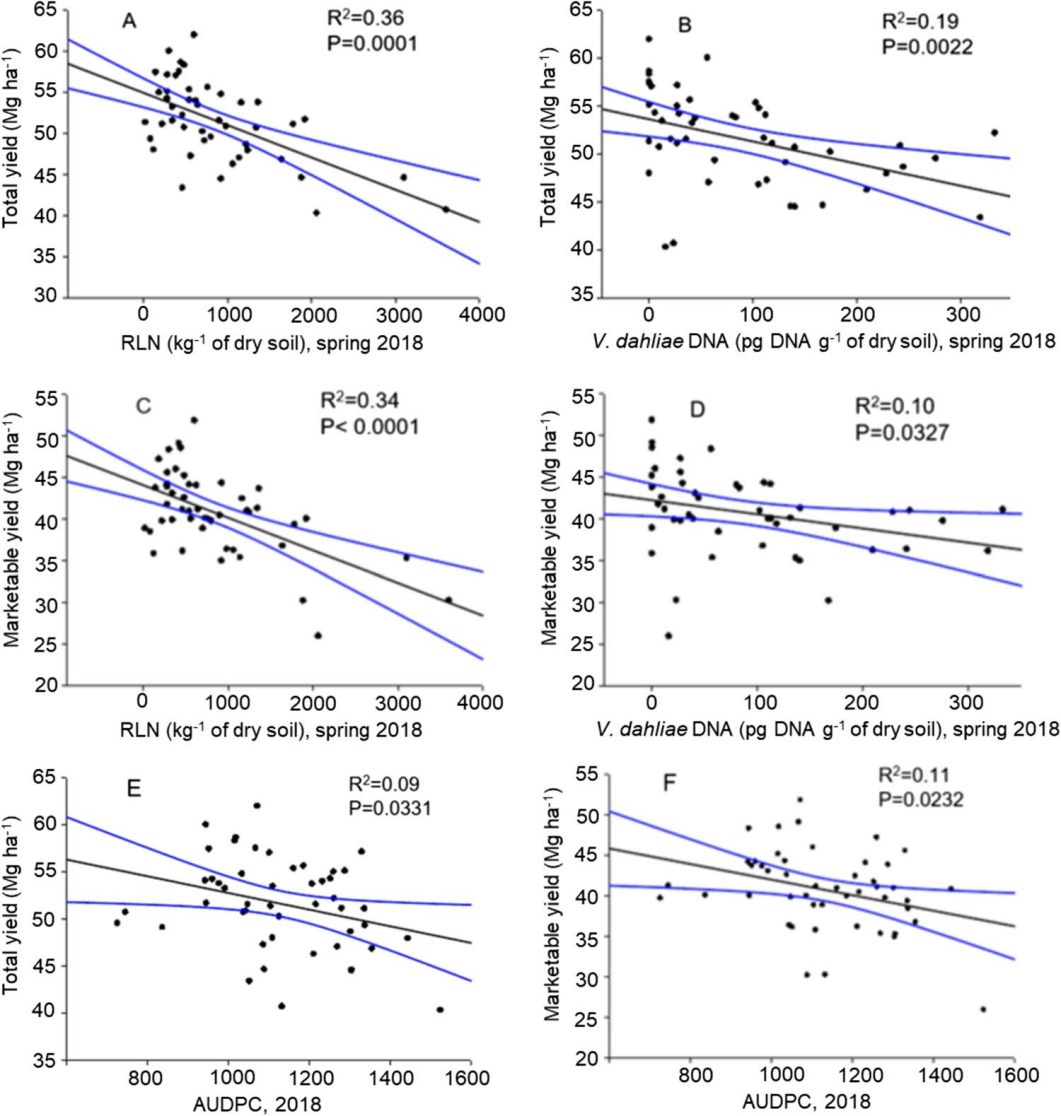
Relationship between the spring root-lesion nematode (RLN) density in the hill, or spring *V. dahliae* density, or PED severity (AUDPC) and the total **A, B, E**, or the marketable tuber yield (Mg ha^−1^) **C, D, F** in 2018  , 95% confidence interval

**Fig. 5:**
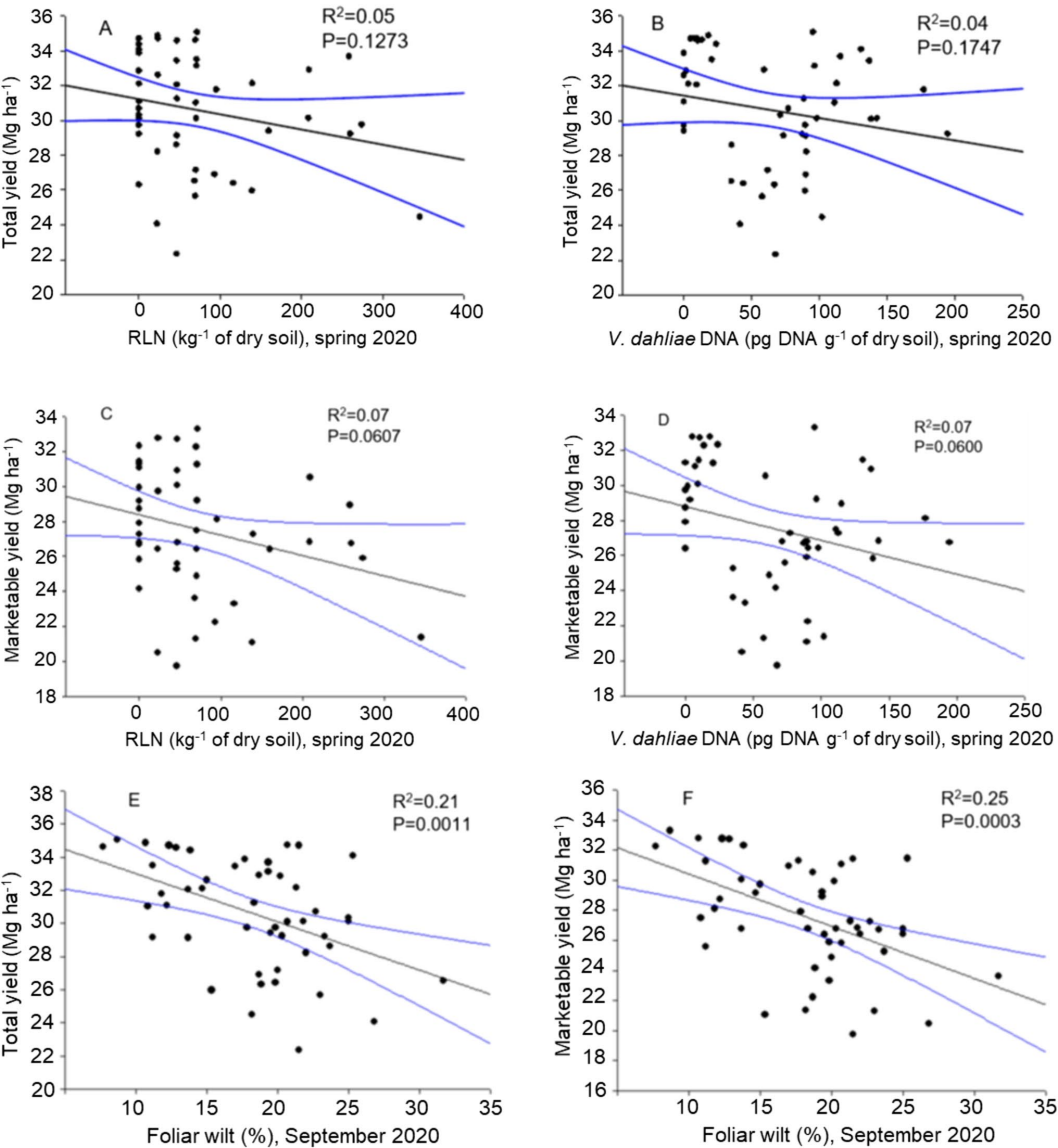
Relationship between the root-lesion nematode (RLN) density in the hill in spring, or *V. dahliae* density in spring, or PED severity (foliar wilt) and the total (**A**, **B**, **E**), or the marketable tuber yield (Mg ha^−1^) (**C**, **D**, **F**) in 2020.  95% confidence interval

In Trial A, RLN density in the hill in spring was significantly negatively correlated with tuber yield, explaining about 36% and 34% of the variance in total and marketable yield, respectively (Fig. 4A, C). Similarly, *V. dahliae* density in spring was significantly negatively correlated with tuber yield, explaining 19% and 10% of the variance in total and marketable yield, respectively (Fig. 4B, D). The PED severity (i.e., AUDPC) in 2018 was significantly negatively correlated with the tuber yield, and explained about 10% of the variance in total and marketable yield (Fig. 4E, F). In Trial B, the RLN and *V. dahliae* densities were not significantly correlated with tuber yield (Fig. 5A, B, C, D), but the PED severity (% foliar wilt) was significantly negatively correlated with tuber yield, explaining 21% and 25% of the variance in total and marketable yield, respectively (Fig. 5E, F).

## Discussion

This study examined the effect of biofumigation on population density of *Pratylenchus* spp. and *Verticillium* spp., PED severity and potato tuber yield in comparison with chloropicrin fumigation and with conventional practice under rain-fed potato production in Eastern Canada. The trials were conducted at a field scale using grower practices on two commercial fields which the grower planned to fumigate due to a known PED issue. Biofumigation was done using ‘Centennial Brown’ brown mustard, a high glucosinolate content and affordable mustard cultivar, with two crop cycles in a growing season to maximize the biofumigation effect on disease suppression and yield improvement, and the soil health benefit from incorporation of two green manure crops. Fumigation followed what is standard practice in NB which is banded application in potato hills to target the volume of soil most critical for potato production, and to reduce cost of fumigant application and environmental footprint.

There was a beneficial potato yield response to biofumgation in Trial A but not Trial B. Extreme drought conditions in 2020 limited potato crop growth and yield, and made it more difficult to detect any beneficial effect of biofumigation on potato yield in Trial B. Biofumigation did not significantly reduce visual symptoms of PED in either trial. Although a significant negative relationship between disease severity and tuber yield was observed, the relationship was weak and therefore the severity of visual symptoms of PED was not necessarily a good indicator of the tuber yield in this study. Biofumigation significantly decreased RLN population density in both trials, but did not significantly decrease *V. dahliae* population density in either trial. This suggests that any yield benefit from biofumigation was likely due primarily to suppression of RLN. The lack of a yield benefit from biofumigation in Trial B may therefore also reflect generally lower RLN population density prior to potato planting for Trial B than for Trial A. Dry soil conditions at the time of mustard incorporation in both trials in this study would be expected to decrease the efficacy of the biofumigation process (Mattner et al., 2008; Omirou et al., 2013). As a result, a more beneficial response to biofumigation may be expected under more favorable environmental conditions, or where irrigation can be used to wet the soil prior to mustard incorporation.

The response to biofumigation in this study is consistent with previous studies. Significant reductions of *P. penetrans* (66–74%) were reported using *B. juncea* seed meal and bran soil amendments as biofumigant prior to potato, strawberry, and maize planting in a greenhouse trial (Yu et al., 2007). Larkin et al. (2011) using a Caliente 119 mustard blend, and Hartz et al. (2005) using *Brassica napus, B. juncea,* and *S. alba* as well as a Caliente blend, demonstrated that mustard crops were ineffective in suppressing Verticillium wilt and that yield improvement was inconsistent in different trials. The efficacy of mustard biofumigation on population densities of soil-borne pathogens can be affected by glucosinolate content of the mustard residues and the soil characteristics (e.g., soil moisture, temperature and pH) at the time of plant residue incorporation into soil (Bending & Lincoln, 2000; Kirkegaard et al., 1993, 1998; Wood et al., 2017). It is also possible that it may require a higher dose of the biocidal products to effectively suppress or kill the *Verticillium* spp. than to kill the RLN (Davis, 1985; Wood et al., 2017).

Chloropicrin fumigation significantly increased potato yield in both trials. Fumigation significantly decreased visual symptoms of PED in Trial B but not Trial A, again indicating that visual symptoms are not necessarily a good indicator of the yield potential in this study. In both trials, the RLN population was suppressed in the hill, where the fumigant was injected, but not in the furrow. Similarly, the *V. dahliae* population density in the hill, but not the furrow, was suppressed prior to potato planting in both trials. This effect of fumigation on *V. dahliae* population density did not persist, however, and did not differ from the control treatment at potato harvest in either trial. The yield benefit from fumigation was therefore likely a result of suppression of both RLN and *V. dahliae* population density, as well as other soil-borne pathogens that were not determined in this study.

The suppression effect of chloropicrin on RLN and *V. dahliae* population densities was in agreement with what was achieved with other fumigants or nematicides (Rowe & Powelson, 2002). Larkin et al. (2011) reported that the microsclerotia of *V. dahliae* and Verticillium wilt severity were significantly decreased after soil fumigation with metam sodium (Vapam) compared to the control in Maine, United States, and Molina et al. (2014) found that Vapam fumigation reduced soil propagule density of *V. dahliae* but did not reduce disease severity in Manitoba, Canada. The effects of fumigants or nematicides on RLN suppression varied with the kind and amount of fumigants or nematicides, application methods, potato varieties, and growing conditions after fumigation (Kimpinski & Sanderson, 1989; Miller & Hawkins, 1969). Potato yield improvement by fumigation, including using chloropicrin, was inconsistent across studies (Ben-Yephet et al., 1983; Bittara et al., 2017; Davis et al., 1986; Hutchinson, 2005; Larkin et al., 2011; Molina et al., 2014).

In this study under the rain-fed potato production in NB, the yield benefit from biofumigation over a two-year rotation cycle was similar to or less than that from chloropicrin fumigation. The less consistent performance of biofumigation compared with fumigation was attributed primarily to two factors. First, the benefit of biofumigation is more dependent on environmental conditions, particularly in rain-fed production where dry soil conditions can limit the efficacy of the biofumigation reaction and limit retention in the root zone of the volatile gas produced (Mattner et al., 2008; Omirou et al., 2013). Environmental conditions can also influence the quantity and glucosinolate concentration of mustard biomass produced. Second, biofumigation suppressed RLN counts but not *V. dahliae* population density, whereas chloropicrin fumigation suppressed both RLN and *V. dahliae* population density. Thus, when considering potato yield response and pathogen suppression in the short term, biofumigation is beneficial but is not as effective as chloropicrin fumigation.

While the current study examined the response to biofumigation on a two-year rotation cycle, it did not consider the longer-term implications of this practice. Biofumigation provides large inputs of plant biomass (approximately 6.2 Mg ha^−1^ dry biomass in two cycles of mustard crops in 2017 in the current study) as a green manure which can increase the quantity and quality of soil organic matter and enhance nutrient cycling, thereby improving soil health (Cherr et al., 2006), and can suppress a range of plant pathogens (Hao et al., 2003; Kirkegaard & Matthiessen, 2004; Morales-Rodríguez et al., 2016; Ngala et al., 2015). In an incubation study, there was little evidence of an adverse effect of biofumigation with mustard residues on nitrifier or denitrifier gene abundance (Sennett et al., 2021) or on microbial diversity (Sennett, 2022). In comparison, chloropicrin fumigation resulted in reduction of *V. dahliae* population density in the hill prior to potato planting, but it was short-lived, and increased to values similar to the control treatment over just one potato growing season. Pathogen suppression by chloropicrin was observed only in the treated hill as the furrow was not treated, whereas the biofumigation suppressed RLN in both the hill and furrow because the hill and furrow were formed after biofumigation. Consequently, fumigant application may be required prior to each potato growing season to achieve the treatment benefit (Davis, 1985). In addition, chloropicrin in an incubation study reduced nitrifier or denitrifier gene abundance (Sennett et al., 2021) and resulted in a substantial and persistent change in the soil microbial community (Sennett, 2022). Fumigation with chloropicrin may, therefore, be at the risk of human health, have an adverse effect on soil health and the environment, as well as have a high operational cost (Bünemann et al., 2006; Sande et al., 2011).

Evidently, biofumigation provides growers with an option in sustainable management of soil-borne diseases, particularly in organic farming and fields with high RLN population density, while maintaining or improving soil health (Gamliel & van Bruggen, 2016; Rajagopal et al., 2019). However, neither biofumigation nor fumigation used alone may be sustainable in the two-year potato rotations commonly used in New Brunswick, and additional beneficial practices are required to sustain productivity in the long-term.

The present study for the first time demonstrated at the commercial field scale that biofumigation was as effective as chloropicrin fumigation in suppressing RLN prior to potato planting and sustaining RLN at low levels after potato growing season relative to the control, as well as improving potato yield, in eastern Canada. Biofumigation was, however, ineffective in suppressing *V. dahliae*. Mustard biofumigation can be a promising alternative to fumigation, particularly in fields with a high RLN population but relatively low *Verticillium* population density, considering its beneficial effect on suppressing various plant pathogens, improving soil health, and low risks to field operators and the environment. However, multiple years of biofumigation alone or in combination with other disease-suppressive practices may be needed to substantially boost biofumigation efficacy for reducing PED or other soil-borne diseases in heavily infested fields. Effective management of PED requires an integrated disease management strategy, and there is a need to further examine the compatibility of biofumigation with other components of an integrated disease management system, to investigate the long-term effect of biofumigation in potato production systems, and to study the interaction between drought and biofumigation on PED disease severity and yield responses under challenging climatic conditions.
